# Lag-structure in fMRI across Three Psychiatric Groups: State-dependency and Clinical-behavioral Correlates

**DOI:** 10.1007/s10548-025-01148-5

**Published:** 2025-09-26

**Authors:** Livio Tarchi, Stefano Damiani, Paolo La-Torraca-Vittori, Giovanni Castellini, Pierluigi Politi, Paolo Fusar-Poli, Valdo Ricca

**Affiliations:** 1 Psychiatry Unit, Department of Health Sciences, University of Florence, Viale Pieraccini, 6, 50139 Florence, FI Italy; 2 Department of Brain and Behavioral Sciences, University of Pavia, Viale Golgi 19, 27100 Pavia, PV Italy; 3 Department of Psychosis Studies, Early Psychosis: Interventions and Clinical-detection (EPIC) Lab, Institute of Psychiatry, Psychology & Neuroscience, King’s College, London, UK; 4 Maudsley Biomedical Research Centre, National Institute for Health Research, London, UK; 5 Psychiatry Unit, University Hospital of Florence, viale della maternità, Padiglione 8b, 50134 Florence, FI Italy

**Keywords:** Neurophysiology, Context-Dependency, Cortical propagation, Network dynamics, Time delay

## Abstract

fMRI measures beyond zero-lag functional connectivity could serve as useful tools for understanding the distinct spatio-temporal dynamics characterizing psychiatric conditions. Therefore, the primary objective was to investigate whether and how state-dependence influences lag-structure in healthy controls (*n* = 95). Moreover, the study aimed to explore clinical-behavioral correlates of state-dependent lag-structure in three groups of psychiatric patients (35 ADHD, 38 Bipolar Disorder and 23 Schizophrenia patients, diagnosed according to DSM-IV-TR). Lag-structure was computed from cross-correlation coefficients in resting-state and stop-signal scans. Between and within-group differences were compared through non-parametric tests. Correlations with clinical-behavioral parameters were evaluated using linear regressions (Brief Psychiatric Rating Scale - BPRS, task reaction time). Compared to healthy controls, lag-structure within default-mode, executive-control and salience networks was generally increased in ADHD (min Z-score − 3.983), generally decreased in Schizophrenia (min Z-score − 3.716) and mixed increased/decreased in Bipolar patients (min Z-score − 3.912, max 4.739). Widespread state-dependent reductions of lag-structure were observed across all groups from rest to task (max Q-statistics: healthy controls 58; ADHD 22; Bipolar 26; Schizophrenia 17). Correlations with clinical-behavioral features (BPRS, reaction time) were positive in the executive-control network and negative in the bilateral thalamus for ADHD; negative in the cerebellum for Schizophrenia; positive in the right temporal gyri, amygdala, hippocampus, cerebellum for Bipolar Disorder (*p* = 0.05). In summary, according to these preliminary results, differences in lag-structure in comparison to healthy controls may be described as progressively increased in magnitude from ADHD to Bipolar Disorder and Schizophrenia, with specific clinical and behavioral correlates according to each diagnostic group.

**Supplementary Information:**

The online version contains supplementary material available at 10.1007/s10548-025-01148-5.

## Introduction

Several meta-analyses have described altered intrinsic brain activity across mental disorders (Cortese et al. 2012; Gong et al. 2020; Merola et al. 2024; Samea et al. 2019). Nonetheless, growing criticism has risen towards the high heterogeneity of these findings (Samea et al. 2019). This heterogeneity may stem from multiple factors, each exerting variable influence and each requiring different mitigation strategies. First, neurofunctional measures may need to account for more complex patterns of intrinsic brain activity, beyond widespread approaches based on temporal correlation among distant brain-regions (i.e. functional connectivity - FC), possibly providing more valuable insight into underlying differences between diagnostic groups and healthy controls (HCs, Jin et al. 2017; Li et al. 2021). Second, state-dependent brain reconfigurations across different mental states, such as changes occurring when shifting from rest to task conditions, should be explored as possible crucial markers of the behavioral responses enacted to real-world contextual changes (Mennes et al. 2010; Zhang et al. 2023).

### Lag-structure as a Proxy of the Complex, Intrinsic Brain Activity

As previously mentioned, analyses of intrinsic brain activity may need to explore approaches beyond temporal correlation alone. Indeed, intrinsic brain activity can be considered not only as a function of static or unimodal FC, but also as the result of interactions between neural configurations. In turn, these different neural configurations may better reflect the complexity of mental states underlying psychological phenomena or mental disorders (Bandettini 2020; Calhoun et al. 2014; Preti et al. 2017). However, the search for neurobiological correlates of intrinsic brain activity has been hindered by important limitations (Lurie et al. 2020). For instance, FC, the main measure describing the synchronization of Blood-Oxygen-Level-Dependent (BOLD) fluctuations across different brain regions, has shown low inter-scan or inter-site reliability, as well as high individual temporal variance (Noble et al. 2019). Similar issues have been found for dynamic connectivity (Zhang et al. 2018, 2022), which refers to FC observed as a function of time using sliding-windows or frame-wise parcellations of the fMRI timeseries (Matsui et al. 2019; Preti et al. 2017). Both classic (“static”) and dynamic approaches for FC consider only aggregated co-activation patterns along the same timepoint, therefore neglecting the possible delay or lag in the interaction between brain regions (i.e. FC measures are characterized by “zero-lag”).

In an attempt to better understand the real-world relationship between neural configurations and mental states, novel approaches have been developed, which consider FC beyond zero-lag alone (Bolt et al. 2022; Liu et al. 2018; Mitra et al. 2017; Pines et al. 2023). In summary, according to these perspectives, FC may be better conceptualized as under the influence of delayed patterns of synchrony resulting from the propagation of brain signals in the span of seconds (Mitra et al. 2014). As timeframes of 1–2 s can be efficiently explored with fMRI, researchers have differentiated zero-lag co-activation patterns from higher-order dynamics involving functional delays (i.e. from here on, “lag-structure”). Importantly, in contrast to zero-lag, preliminary analyses indicated a high test-retest reliability for lag-structure measures (Choe et al. 2017).

### State-dependence and Contextual Experiences

Another unexplored aspect of brain dynamics is how intrinsic lag-structures may reconfigure in response to variations in mental states. For this purpose, it is useful to first define the term *state-dependence*, which encompasses the sum of reconfigurations occurring in the brain when transitioning from rest to task conditions (Bradley et al. 2022; Lynch et al. 2018). Previous results showed that state-dependent reductions in local connectivity correlate with symptom severity in Schizophrenia, and state-dependent dynamics associate in a diagnostic-specific manner with performance (Damiani et al. 2022).

Based on these premises, three open questions gain relevance. First, whether functional connectivity beyond zero-lag is associated with alterations within specific diagnostic groups of psychiatric disorders (Schizophrenia, Bipolar Disorder, and Attention Deficit Hyperactivity Disorder – ADHD). Second, whether lag-structure in HCs also exhibits state-dependence from rest to task conditions. Finally, whether state-dependent reconfigurations could be associated with clinical and behavioral factors in psychiatric conditions, and, thus, shed light into the observed heterogeneity between patients within the same diagnostic group (Segal et al. 2023).

### Aims

The primary aim of the present study was to investigate whether lag-structure undergoes functional reconfigurations from rest to task conditions. We expected to observe reduced lag-structure during the task compared to rest due to the faster reaction times imposed by the task condition. Secondly, the present study aimed to test whether state-dependent lag-structure reconfigurations were correlated with clinical and behavioral outcomes. We expected that greater reductions of the lag-structure would correspond to better performance during the task, and to lower clinical severity.

## Materials & Methods

### Sample

The data supporting the current analyses were collected from the UCLA Consortium for Neuropsychiatric Phenomics (Poldrack et al. 2016). All subjects were right-handed and aged between 21 and 50 years old. Participants with lifetime diagnoses of Schizophrenia or Other Psychotic Disorder, Bipolar I or II Disorder, Substance Abuse or Dependence, Anxiety Disorder (Obsessive Compulsive Disorder, Panic Disorder, Generalized Anxiety Disorder, Post-Traumatic Stress Disorder), suicidality, or current Major Depressive Disorder were excluded from the group of HCs. All controls were screened for sub-threshold ADHD - defined as having more than three symptoms of inattention or hyperactivity/impulsivity in either childhood or adulthood (Poldrack et al. 2016). Diagnoses were based on the Diagnostic and Statistical Manual of Mental Disorders Fourth Edition - Text Revision (DSM-IV-TR; American Psychiatric Association 2000), following a structured clinical interview (SCID-I; First and Gibbon 2004) supplemented by the Adult ADHD Interview (derived from the Kiddie Schedule for Affective Disorders and Schizophrenia, Present and Lifetime Version; Kaufman et al. 1997). Each of the three patient groups (Schizophrenia, Bipolar Disorder, ADHD) excluded anyone with any other diagnosis. The phase of illness for patients with a diagnosis of Bipolar Disorder was assessed at the time of recruitment using the Brief Psychiatric Rating Scale (BPRS).

### Study Design

Participants were instructed to remain relaxed and keep their eyes open during the fMRI scans. The resting-state fMRI scan lasted 304 s (152 timepoints, repetition time 2 s), and no stimulus was presented during resting-state. For conducting analyses on the secondary aims, the stop-signal task was chosen, as its design implies a constant recruitment of attentive resources across the whole scan-time. This characteristic made the stop-signal task a continuous state ideal to be compared to rest. The stop-signal task run lasted 386 s (193 timepoints, repetition time 2 s). Participants were instructed to respond as fast as they could after a ‘go’ stimulus was presented on the computer screen, except for the subset of trials where the ‘go’ stimulus was paired with a ‘stop’ signal, requiring them to withhold their response.

Performance was then measured through the Stop-Signal Reaction Time (SSRT), with longer SSRTs corresponding to worse performances. Further details on the task condition, and on how SSRT was determined, can be found in the Supplementary Materials (eMethods 1). The mean stop-signal reaction time (SSRT) was used as a proxy to investigate the overall performance during the task as a measure for behavioral outcomes (see eMethods 1). Correlations were also explored between state-dependent lag-structure and total score of the BPRS – a brief tool measuring psychopathological domains such as anxiety, depression, emotional withdrawal, psychotic symptoms (hallucinations, disorganized thought, blunted affectivity), hyperactivity and hostility (Overall and Gorham 1962), as a measure for clinical features of individual patients.

All MRI scans were collected on one of two 3 T Siemens Trio scanners. A T2*-weighted echo-planar imaging (EPI) sequence was acquired for both the resting-state and task condition, with the following parameters: slice thickness 4 mm, voxel size 3 × 3 × 4 mm, matrix 64 × 64, echo time 20ms, flip angle 90°. Additionally, a high-quality anatomical scan was acquired for each participant, with the following characteristics: MPRAGE sequence, TR 2.26 ms, slice thickness 1 mm. To ensure anonymity, all anatomical scans were defaced.

### Preprocessing

fMRI data preprocessing steps were implemented in AFNI (Cox 1996; Cox and Hyde 1997; Taylor et al. 2018). The first 4 frames of each fMRI run were removed in order to discard the transient effects in amplitude observed until the MRI scanner achieves steady-state (Caballero-Gaudes and Reynolds 2017). Slice timing correction (Konstantareas and Hewitt 2001) and despike methods (Satterthwaite et al. 2013) were then applied.

The structural and a functional reference image (given by the median of each timepoint) were firstly co-registered (Saad et al. 2013), in order to remove translational or rotational differences, through a rigid-body transformation of the structural image (*3dAllineate* by AFNI, cost function given by the local Pearson correlation). A rigid-body alignment was also computed for each timepoint in the functional dataset to the structural image. Six motion parameters were estimated for each individual scan. The anatomical image was then warped using the Montreal Neurological Institute standard space template (MNI152 “2009c”). Volume registration was used to align the functional data to the base volume, warping it to the stereotactic space of choice.

Spatial blurring was performed, with a kernel of full width at half maximum of 6 mm. Bandpass (0.01–0.1 Hz) was also performed (Shirer et al. 2015). Tissue segmentation was performed with the AFNI 3dSeg command, in order to obtain an individual mask of gray matter, white matter and cerebro-spinal fluid. Each of the voxel time series was then scaled to have a mean of 100. To control for non-neural, physiological noise, regression based on the rigid body motion parameters and their derivatives was applied, as well as mean time series from cerebro-spinal fluid masks (Fox et al. 2005) eroded by one voxel (Vovk et al. 2011). Regression of white matter artifacts was performed through the fast ANATICOR technique as included in AFNI (Jo et al. 2010). Censoring was not performed in order to preserve the time continuity needed to compute the lag-structure. Nonetheless, to further improve motion correction, subjects with excessive motion were excluded, excessive motion was defined as > 2 mm translation or > 2° rotation. Moreover, subjects with a mean Framewise Displacement (FD), a measure of head movement during scan, calculated as the sum of absolute values of realignment estimates at each timepoint, over 0.5 mm were excluded (Power et al. 2014).

Fifteen HCs, one participant with ADHD, one participant with Bipolar Disorder and nine individuals with Schizophrenia were excluded due to motion. Participants with Schizophrenia were overrepresented among excluded participants (Chi-Square *p* = 0.002). Among included participants, after quality control and exclusion due to motion, patients with Schizophrenia still reported slightly higher mean FD per run in comparison to HCs (rest: 0.229 ± 0.068 vs. 0.183 ± 0.055, task: 0.181 ± 0.052 vs. 0.147 ± 0.049; see Supplementary Figure S1a and S1b).

Task stimuli were not regressed from the task condition, as the process of eliminating task-evoked responses from correlation analyses was previously found to have only minimal effect on results pertaining to functional connectivity (Cole et al. 2014). Previous findings also show how the difference in functional connectivity between rest and task conditions is mostly independent from the regression of task-evoked responses (Lynch et al. 2018).

### Lag-structure

The lag-structure of each individual scan was measured using the AFNI command “3ddelay”, with the gray matter mask representing the global signal average from which the cross-correlation coefficients are derived (Cox 1996). The zero-lag FC of each voxel is the correlation between the voxel timeseries and the global signal at the same time point. To go beyond zero-lag, FC for each voxel is initially calculated as the cross-correlation coefficient between the voxel timeseries as a seed and global signal at lag = 1 (equivalent to 1 TR = + 2 s). The same procedure is carried out for all increments of lag.

While period signals could show multiple symmetrical cross-correlations (Mitra et al. 2017), fMRI time series are aperiodic in nature and show one extremum in cross-correlation functions (He 2014; Mitra et al. 2017). Therefore, the strongest cross-correlation coefficient indicates at which timepoint, or lag, each voxel shows the highest concordance with global signal. Higher lag-structure hence corresponds to an increased time span at which the strongest cross-correlation coefficient is measured. The maximum upper limit for lag was set at 15 timepoints (30 s) for both the resting-state and task scan, as computing lags higher than 10% of the overall number of timepoints is not currently advised (Saad et al. 2001, 2003).

In order to further enhance the reproducibility of the procedure, and robustness of results, the cross-correlation was not derived by parabolic interpolation (Mitra et al. 2017), but was limited to the values observed in the fMRI timeseries itself. Moreover, the voxelwise output of “3ddelay*”* was also thresholded for a minimum cross-correlation coefficient of 0.10, a two-tailed voxelwise p-value of p = 0.01, a minimum cluster size of 30 voxels (three nearest neighbors, NN). Results were further masked in order to represent only results pertaining to the gray matter. A post-hoc analysis of mean correlation coefficients per run was conducted.

Lag-structure in the resting-state had an average rho of 0.38 ± 0.3 (min 0.32, max 0.52), while task 0.34 ± 0.3 (min 0.28, max 0.54). A graphical representation of mean correlation coefficients by clinical group was offered in the Supplementary Materials as Figure S2a and S2b.

### Statistical Methods

In order to better describe the sample, the lag-structure at resting-state was first compared between groups. After considering previous works showing strong right-skewed distributions, with most voxels exhibiting low delay, and a small portion of voxels characterized by higher delay (Mitra et al. 2014), normality was not assumed for lag-structure. For this reason, nonparametric Mann–Whitney U tests were employed. Mean lag values, where cross-correlation coefficients reached the maximum, were compared between each diagnostic group (ADHD, Bipolar Disorder, Schizophrenia) and HCs. An a priori threshold of *p* = 0.05 and a minimum cluster size of 30 voxels was chosen for statistical significance. Clusters of results were reported according to the N27 functional atlas (Eickhoff et al. 2005, 2006, 2007), and interpreted as part of a brain network as described by Shirer et al. (2012).

In accordance with the primary aims of the study, state-dependence was computed within groups. As the lag-structure is not posited to follow a normal distribution, Friedman tests for paired samples were employed. As a correction for multiple comparisons is not compatible with the estimation of nonparametric tests in fMRI (Jr 1997; Lehmann and D’Abrera 2006; Noether and Dueker 1990; Ward 1997), previous literature adopted a significance threshold for Friedman tests in fMRI at *p* = 0.05 (Audoin et al. 2003). To enhance reliability of the current study, a more conservative approach at *p* = 0.01 was adopted for comparisons within-subjects. Results were further thresholded for a minimum cluster size of 30 voxels (as calculated by third-nearest neighbor clustering). Permutation tests to correct for multiple comparisons could not be performed, due to the violation of the underlying assumption of exchangeability (Good 2002) when thresholding individual results according to statistical significance.

Parameters covarying with the effect of state-dependence (mean SSRT, total BPRS score) were explored through the AFNI command *3dttest++* (Cox 1996). The level of significance for covariates was set at *p* = 0.05 (minimum cluster size 30, NN 3).

### Control Analyses

Between-group differences were also explored at *p* = 0.01, as secondary sensitivity analysis. The possible confounding effect of the global signal on group comparisons was assessed, repeating both within (state-dependence) and between-groups (HC vs. clinical groups) comparisons after adding Global Signal Regression (GSR) to the pre-processing of individual scans. The possible role of motion in determining state-dependent changes in lag-structure was explored estimating the correlation coefficients of voxel-wise state-dependent lag values with mean FD in each run.

## Results

### Descriptive Statistics

A total of 95 HCs, 35 patients with ADHD, 38 patients with Bipolar Disorder and 23 patients with Schizophrenia constituted the final sample. Males were overrepresented across subgroups (51.57% for HCs, 51.42% for patients with ADHD, 55.26% for Bipolar Disorder, 69.56% for Schizophrenia). A summary of demographics is provided in Table 1. Mean lag-structure values per group (in seconds) during resting-state were also graphically represented in the Supplementary Materials as Figure S3.

**Table 1:** Sample descriptives

	HC	ADHD	BIP	SCH
N	95	35	38	23
Age	30.9 ± 8.56	31.2 ± 10.3	34.3 ± 9.38	35.1 ± 9.21
Sex	46 females 49 males	17 females 18 males	17 females 21 males	7 females 16 males
Mean FD resting state	0.182 ± 0.055	0.180 ± 0.064	0.207 ± 0.079	0.227 ± 0.066
Mean FD stopsignal	0.146 ± 0.050	0.153 ± 0.045	0.166 ± 0.063	0.183 ± 0.055
SSRT (seconds)	213 ± 40	224 ± 34	219 ± 46	249 ± 37
Mean BPRS total score	/	37.31 ± 6.64	43.18 ± 10.33	53.82 ± 15.06

Note: minimum age in the sample 21 years old, maximum age 50 years old.

HC: healthy controls

BIP: patients with Bipolar Disorder

SCH: patients with Schizophrenia

The group of patients with ADHD showed increased lag-structure in areas pertaining to the posterior central executive network. Three areas were found localized respectively in the middle occipital and temporal gyrus (Z-score − 3.983), superior and inferior parietal lobule (Z-score − 3.580) and in the left calcarine and lingual gyri (Z-score − 3.034). The remaining surviving clusters showed reduced lag-structure compared with HCs, mainly in the salience, anterior central executive and default networks. These were localized in the bilateral dorsolateral prefrontal cortex, insular lobes, cerebellum (crus I and II), right anterior cingulate, right superior medial gyrus, right middle orbital gyrus, left middle and inferior temporal gyri, right posterior cingulate cortex (max Z-score 4.431, min Z-score 3.210).

Patients with Bipolar Disorder also exhibited reduced lag-structure at rest compared with HCs in the majority of areas, also pertaining to the central executive, salience and default networks (bilateral calcarine gyri, right anterior cingulate, right superior medial gyrus, right lingual gyrus, right thalamus, bilateral superior temporal gyrus, bilateral middle orbital gyrus; max Z-score 4.739, min Z-score 3.868). Nonetheless, three clusters showed increased lag-structure, namely the left and right cerebellum (lobule VI, Z-score − 3.912 and − 3.010 respectively) and the right cerebellar tonsil (Z-score − 3.183).

By contrast, patients with Schizophrenia generally exhibited increased lag-structure compared with HCs in several areas pertaining to the posterior central executive and default networks (right anterior cingulate, right middle and superior medial frontal gyri, right middle lateral frontal gyrus, right postcentral gyrus, right precuneus; max Z-score − 3.716, min Z-score − 2.899). However, the right caudate nucleus, right inferior temporal gyrus, left cerebellum (lobule VII) and the bilateral superior medial gyri (max Z-score 3.838, min Z-score 3.098) showed reduced lag in Schizophrenia. Results for *p* = 0.05 were illustrated in the Supplementary Materials as Table S1 and Figure S4a.

### State-dependent lag-structure

**Fig. 1:**
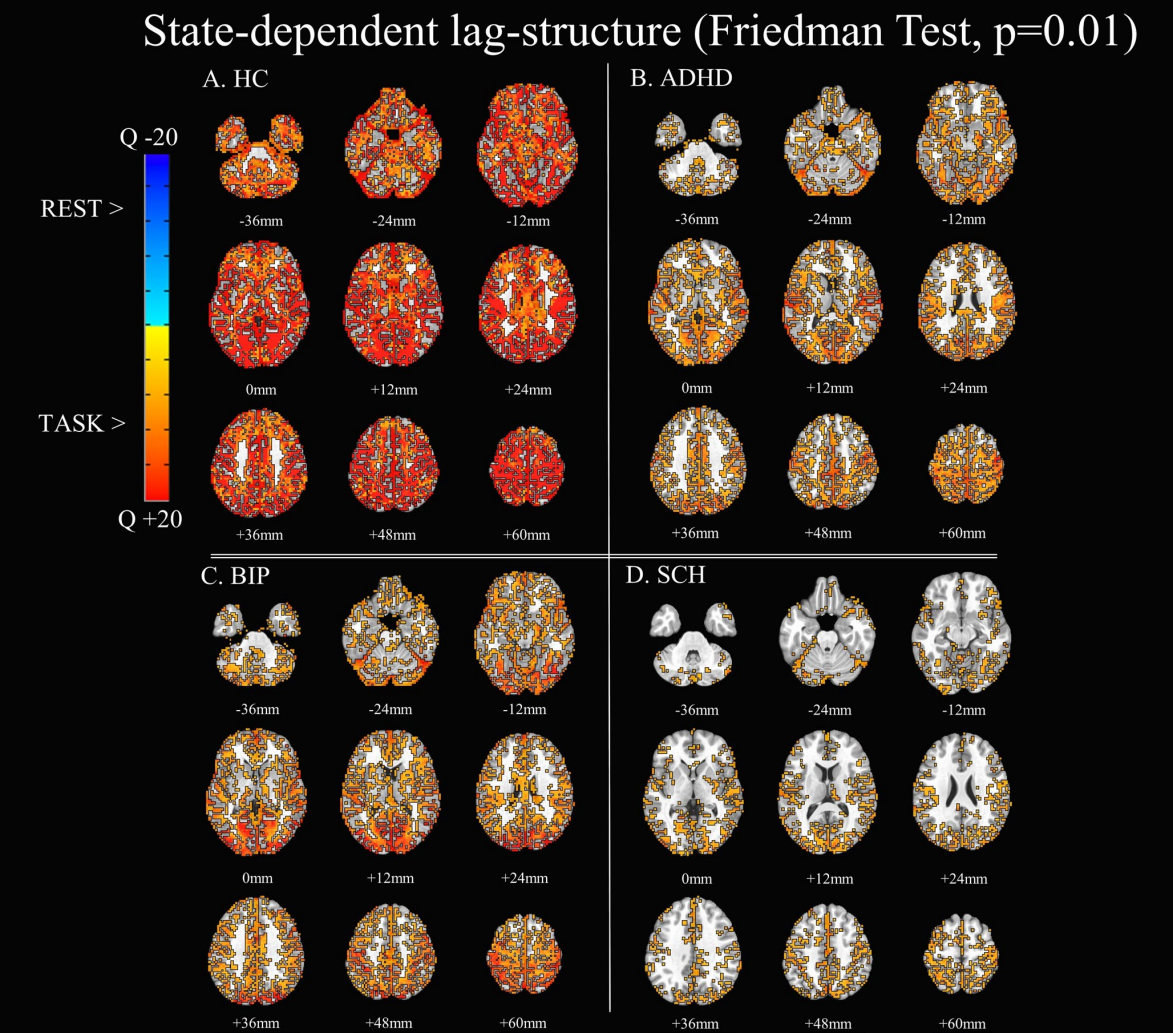
State dependent lag-structure, resting state compared to stopsignal task.In red, higher lag during resting state. In blue, higher lag during stopsignal task (no brain region). Results given by Friedman test for paired observations. Q = Q-statistics **A**. Healthy Controls **B**. ADHD **C**. Bipolar **D**isorder **D**. Schizophrenia

In HCs, lag-structure was reduced across the whole brain in the stop-signal task compared to rest. Reduced lag-structure at task in comparison to rest was also observed across clinical groups. While HCs exhibited a global reconfiguration of lag-structure when switching from rest to task (reduced at task: one cluster of 45386 voxels, max Q-statistics 58, peak localized in the right middle temporal gyrus at x −58.5, y + 67.5, z + 7.5), this effect progressively reduced across clinical groups, with patients with ADHD or Bipolar Disorder at an intermediate level between HCs and patients with Schizophrenia. The lag-structure reconfiguration in ADHD was significant in a single cluster (24316 voxels, max Q-statistics 22, peak localized in the left fusiform and lingual gyri at x + 22.5, y + 64.5, z −13.5). Patients with Bipolar Disorder also showed a single cluster of lag-structure state-dependence (26939 voxels, max Q-statistics 26, peak localized in the right superior occipital gyrus at x −31.5, y + 88.5, z + 28.5). By contrast, patients with Schizophrenia showed reduced state-dependent reconfigurations of lag-structure, with the principal cluster of difference localized in the left lingual gyrus (9830 voxels, max Q-statistics 17, peak at x + 19.5, y + 49.5, z −4.5). All 10 significant clusters in patients with Schizophrenia are listed in Supplementary Table S2. Results by diagnostic group were graphically illustrated in Fig. 1.

### State-dependent lag-structure and Task Performance

Performance was unrelated to lag-structure in HCs. Individuals with ADHD showed a positive correlation between state-dependent lag-structure and reaction time for the posterior central executive network (superior, middle and inferior temporal gyri) and right cerebellum (lobule VI, VII). Stronger correlations are to be interpreted as worse performance.

Patients with Bipolar Disorder showed both positive and negative correlations. Positive correlations were observed in the posterior inferior temporal gyrus and left calcarine gyrus. Negative correlations were observed in the salience network (left lateral medial gyrus, medial superior frontal gyrus, left inferior and middle temporal cortex, left lingual gyrus, bilateral pre- and postcentral gyrus, bilateral rectal gyri), as well as the cerebellum (left lobules IV, V, right lobule VI).

**Fig. 2:**
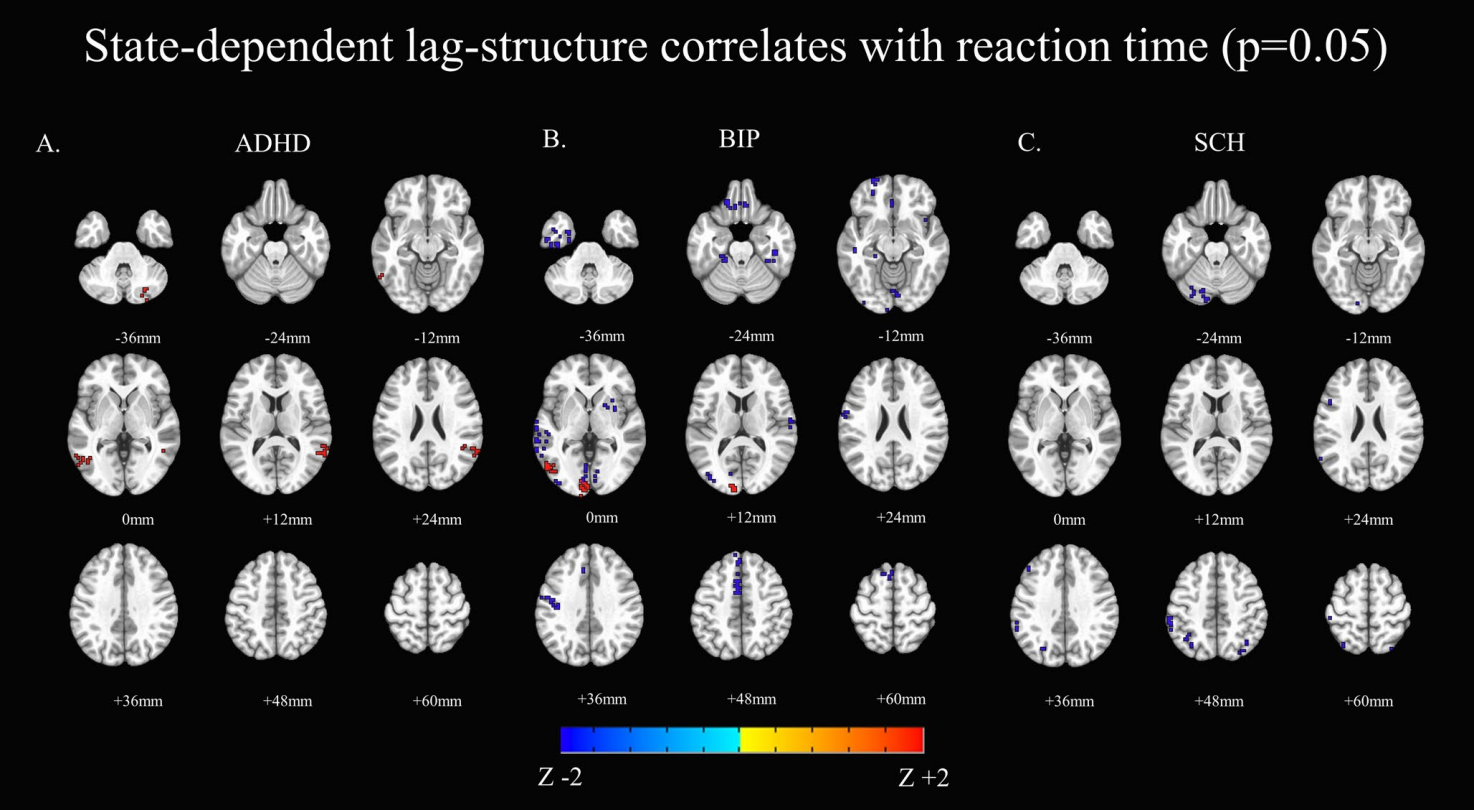
State dependent lag-structure and performance (reaction time) In red, worse performance/higher reaction time. In blue, better performance/lower reaction time. **A**. ADHD **B**. Bipolar Disorder **C**. Schizophrenia

Patients with Schizophrenia showed only negative correlations, in the posterior salience and/or central executive network (left posterior inferior parietal lobule, bilateral posterior superior parietal lobules, left inferior frontal gyrus, posterior inferior temporal gyrus), as well as the cerebellar crus I. Results were summarized in Fig. 2 and in the Supplementary Materials as Table S3.

### Secondary results, Psychopathological Correlates

Severity of overall psychiatric symptoms (total BPRS score) was correlated to state-dependent lag-structure. ADHD individuals showed a positive correlation in lateral cortical structures (left and right inferior frontal lobes, right supplementary motor cortex, right superior frontal gyrus, right inferior, middle and superior temporal gyri, left fusiform gyrus), while negative correlations were found in the basal ganglia (putamen) and in the ventral insular lobes, as well as the frontal opercular areas. ADHD individuals also showed positive correlations in the central executive network (right superior frontal gyri, right supramarginal gyrus, right middle and superior occipital gyri, right pre- and postcentral gyri, left fusiform gyrus, bilateral inferior frontal gyri, bilateral middle temporal gyri). Negative correlations were found in the salience network (both the left and right insular lobes, as well as in the left postcentral gyrus).

**Fig. 3:**
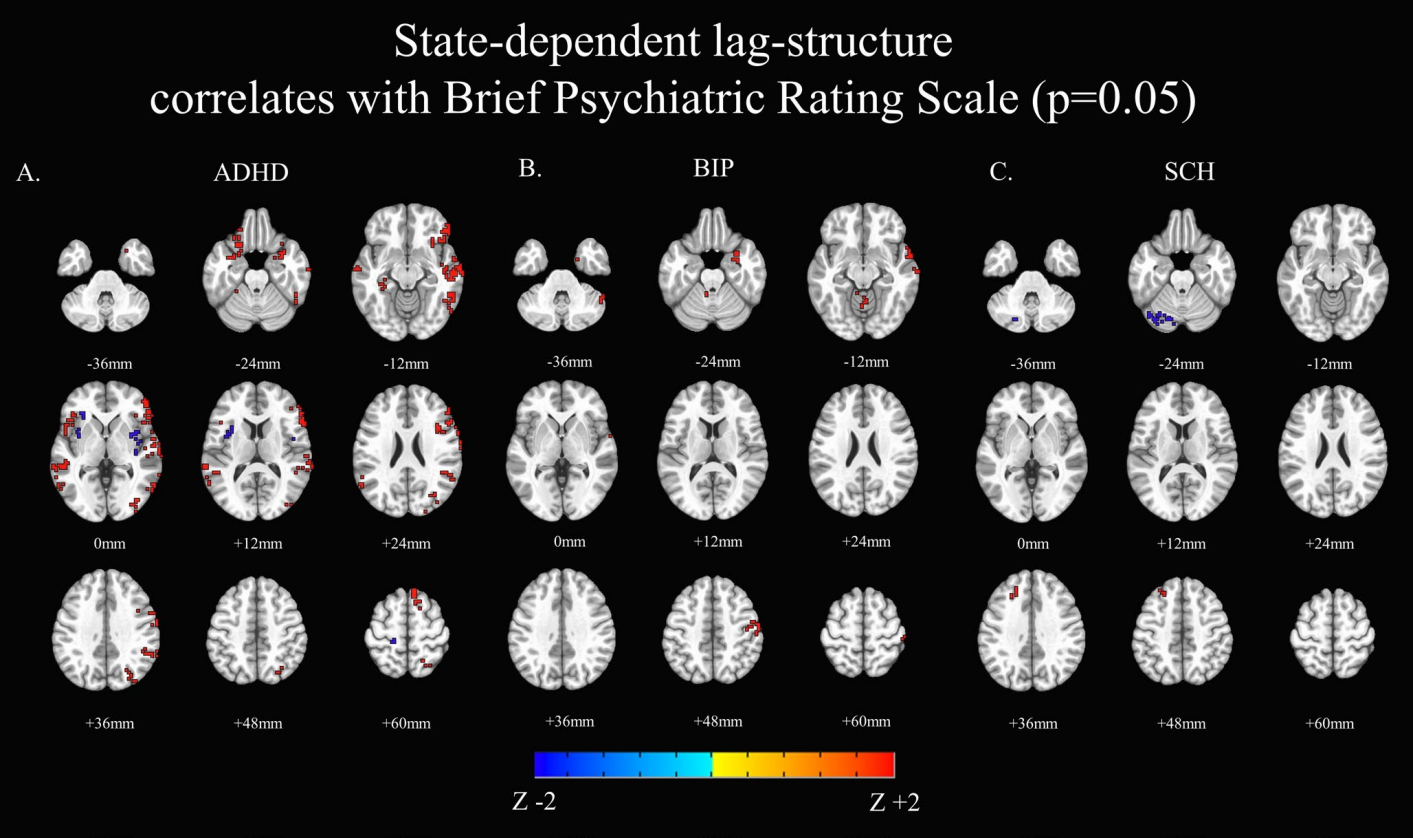
State dependent lag-structure and clinical severity In red, higher severity. In blue, lower severity. **A**. ADHD **B**. Bipolar Disorder **C**.Schizophrenia

The sample of individuals with Bipolar Disorder showed only positive correlations between state-dependent lag-structure and general psychopathology, with surviving voxels in four non-contiguous areas encompassing the right temporal pole, right middle temporal gyrus, right amygdala, right hippocampus, right cerebellum (crus I, II, cerebellar lobules III, IV, V, VII, vermis). The sample of patients with Schizophrenia showed a single area with negative correlations, in the left cerebellum (crus I, lobule VI). Results were summarized in Fig. 3 and in the Supplementary Materials as Table S4.

### Control Analyses

Control analysis at a more conservative statistical threshold of *p* = 0.01 showed no difference of lag-structure at rest between HCs and patients with ADHD or Schizophrenia. Patients with Bipolar Disorder had three surviving clusters at *p* = 0.01 with reduced lag-structure compared with HCs, namely in the right superior medial gyrus (x −10.5, y −49.5, z −1.5, Z-score 3.766), right inferior temporal gyrus (x −52.5, y + 52.5, z −25.5, Z-score 4.040), and the right calcarine gyrus (x −4.5, y + 76.5, z + 10.5, Z-score 3.601).

Sensitivity analyses to test for the role of GSR confirmed that statistically significant results could also be observed when GSR was performed, both for within and between-group comparisons (see Figures S4 and S5, Table S5 in the Supplementary Materials). While state-dependent gradients within-groups, as observed after GSR, were similar to no-GSR results (i.e., highest in HCs, lowest in Schizophrenia, see Figure S5 in the Supplementary Materials), observed clusters were smaller in control analyses. Between-groups comparisons after GSR showed similar results compared to no-GSR for patients with ADHD or Bipolar Disorder. In contrast, increased, rather than decreased, lag-structure was mostly observed for patients with Schizophrenia in comparison to controls after GSR (see Figure S5 and Table S5 in the Supplementary Materials).

No significant correlation between state-dependent lag-structure and motion (mean FD per run) was observed at *p* = 0.05 for HCs, patients with ADHD or Bipolar Disorder. Nonetheless, patients with Schizophrenia showed two small but significant clusters of positive correlation between state-dependent lag-structure and motion during resting-state, one in the left superior temporal gyrus (49 voxels, Z-score 3.281) and the other in the right supramarginal gyrus (31 voxels, Z-score 3.117). See Figure S6 and Table S6 in the Supplementary Materials for detailed information.

## Discussion

Reductions in lag-structure from resting-state to task were observed in all groups. However, the magnitude of this state-dependent reconfiguration was greatest in HCs and progressively reduced in ADHD, Bipolar Disorder and Schizophrenia. As lag-structure represents the latency with which a region maximally connects to the rest of the gray matter, it may quantify how fast local information is globally integrated by the brain. The present results are therefore discussed considering the interplay between default mode, central executive and salience network (Bocharov et al. 2023; Pompilus et al. 2020).

### Lag-structure: Similarities and Divergences across Groups

Compared to HCs, patients with ADHD showed a reduced lag-structure in regions pertaining to the central executive network, patients with Schizophrenia showed mixed alterations in the default mode network (lower lag in the medial prefrontal cortex, higher in the precuneus/angular gyri) and patients with Bipolar Disorder exhibited reduced lag-structure in both executive and default mode networks.

In addition, patients with Schizophrenia showed reduced lag-structure in the cerebellum compared to HCs, while mixed values were observed in patients with Bipolar Disorder. The precuneus, angular gyrus, crus I and crus II have all been associated with mentalization, self-talk and emotional self-experiences (Andrews-Hanna 2012; Van Overwalle et al. 2020). The presence of both differences and similarities between Bipolar Disorder and Schizophrenia in the lag-structure is in line with mounting evidence for a nosological continuum linking these two diagnostic groups, especially when considering patients with Bipolar Disorder also exhibiting psychotic features (Merola et al. 2024; Smeland et al. 2020).

### State-dependent Changes in lag-structure

The group of patients with ADHD exhibited several regions with reduced lag-structure at rest in comparison to HCs. Those areas mainly pertain to the salience network, which has been shown to critically modulate executive and default mode networks acting as a switch between them (Sridharan et al. 2008). Lag-structure was increased in executive control regions and reduced in regions of the default node network, suggesting the presence of imbalances in the dynamic interactions between these two antagonist networks. Lag-structure changes in patients with Bipolar Disorder were intermediate between those observed in HCs and patients with Schizophrenia, further supporting the notion of a spectrum of continuum between these disorders (Argyelan et al. 2014; Haukvik et al. 2018; Murray et al. 2004; Smeland et al. 2020).

The functional integration between the default mode, central executive and salience networks has been previously described both in tasks involving a continuous allocation of attentive resources (Gong et al. 2016; Sridharan et al. 2008) and in those involving discontinuous responses to salient stimuli (Menon and Uddin 2010). The ability to dynamically reconfigure the other brain networks allows the salience network to reallocate attentional resources and efficiently modulate autonomic reactivity and motor responses (Menon and Uddin 2010). Lag-structure, being cross-temporal in nature, may provide a better understanding of dynamic responses during task design, which require both global and local reconfigurations of intrinsic brain activity based on contextual demands. However, to test the hypothesis of a neuronal reallocation of attentional resources during the shift between resting-state and task conditions, future studies might explore other concurrent, alternative measures of information integration in the brain, for instance leveraging recent developments in analyses related to integrated information decomposition (Luppi et al. 2024).

### Lag-structure and Task Performance

The group of patients with ADHD showed worse performance as correlated with the degree of lag-structure state-dependence in areas pertaining to the executive control network. By contrast, the group of patients with Bipolar Disorder showed that worse performance was correlated with the degree of state-dependence in the posterior inferior temporal gyrus and left calcarine gyrus, brain regions where neurofunctional alterations are commonly associated with a diagnosis of mood disorders (Jung et al. 2014; Luo et al. 2022). Finally, the group of patients with Schizophrenia showed worse performance as correlated with the degree of lag-structure state-dependence in the central executive network.

Theoretically, lag-structure indicates the time at which a region is maximally connected with the rest of the brain. Therefore, reduced lag-structure within a region should correspond to shorter times elapsing between its activation and the engagement evoked in the rest of the brain. The authors then propose that greater decreases in the lag-structure from rest to task within a region (i.e., higher state-dependence) may measure its predisposition to interact on a global level. Both the intrinsic lag-structure and its state-dependence were condition-specific, suggesting that each psychiatric group can be characterized by different imbalances in how local signals are integrated at a global level. Future studies might thus explore this specific hypothesis, and whether the integration of global signals may be disrupted across diagnostic groups in psychiatry.

### Clinical Correlates of lag-structure

While widespread associations were found between state-dependent lag-structure and clinical severity in ADHD, more limited evidence was observed for patients with Schizophrenia. Nonetheless, similarly to patients with ADHD, positive correlations between state-dependent lag-structure and general psychopathology were also observed for Bipolar patients. Interestingly, these correlations were observed primarily in brain regions that have been more consistently associated with mood disorders rather than psychotic symptoms (Blond et al. 2012). For instance, the right temporal lobe has been previously associated with rapid mood cycling in Bipolar Disorder, and this brain region was here observed as exhibiting a positive correlation between lag-structure and clinical severity (i.e. higher delay, higher severity). Future studies might explore whether functional alterations in the right temporal lobe may be leveraged as a diagnostic-specific biomarker for Bipolar Disorder. Future studies might also explore whether cerebro-cerebellar connectivity, which here showed divergent patterns of association with clinical features across Bipolar Disorder and Schizophrenia, may also aid in the development of robust diagnostic biomarkers for these disorders, possibly aiming to reach a diagnostic differential between these two clinical entities.

### Limitations

Standard pre-processing measures were used in the present study in order to limit the potential effect of motion on results. While correction for multiple comparisons could not be employed, the cluster-size of primary results strongly contrasts with the null hypothesis (over 9800 voxels for patients with Schizophrenia, 20.000 voxels for patients with ADHD or Bipolar Disorder, over 45.000 voxels for HCs). Nonetheless, future studies might employ random generative models for lag-structure in order to better control for type II statistical errors, in particular for what concerns the regional distribution of the findings.

Control analyses did not find significant differences in state-dependent lag-structure as influenced by motion during the scan, with the exception of two regions in the Schizophrenia sample (one in the left superior temporal gyrus, and the other in the right supramarginal gyrus). However, caution is warranted in general for fMRI studies, as motion has a known influence on results. Indeed, while the current study employed several techniques to correct for non-neural sources of noise during fMRI, future studies might explore whether and how cardiac and respiratory function influence lag-structure, as well as whether case-control differences may survive after further correction for these potential confounders.

Excluding scans characterized by high motion resulted in a significant reduction of the original sample size, especially for the Schizophrenia group. Therefore, caution is warranted in the interpretation and generalizability of current results. Future replication in other samples is then required.

Additionally, the sample of patients with Bipolar Disorder included phases of euthymia, depression, and mania/hypomania. Future studies may be interested in exploring the role of current clinical status on lag-structure for Bipolar Disorder.

GSR results highlighted higher values of lag in patients with Schizophrenia compared to HCs. Such findings have an opposite direction compared to no-GSR results. This is not surprising considering the previous literature on the topic, which found potentially opposite directions of effects in several measures when GSR was applied (Gotts et al. 2013). As lag-structure involves extended timeframes where a global coordination of the brain is expected, it would be more likely for global signal to vehicle significant information rather than noise. However further studies are needed to test the specific role of global signal in lag-structure.

Finally, previous studies showed a role for contextual experiences in influencing fMRI results. This evidence primarily showed diverging fMRI dynamics due to the influence of social interactions prior to scans, the exposure to novel environments (Bocharov et al. 2023; Pompilus et al. 2020) or the overall arousal state of the participant (Raut et al. 2021). How these processes may influence fMRI results is still an evolving topic and should be explored in future studies.

## Conclusions

The present study provides preliminary insight into the neurophysiological mechanisms of state-dependence, beyond zero-lag functional connectivity, and presents preliminary evidence for clinical and behavioral correlates of this underlying neural process for specific psychiatric disorders (i.e. ADHD, Bipolar Disorder, Schizophrenia). The brain-wide state-dependent reconfiguration of the lag-structure in HCs, and the differences observed across different psychiatric conditions, suggest that this measure may shed new light on the spatiotemporal dynamics of fMRI, especially in relation to potential alterations within psychiatric disorders.

## Supplementary Information

Below is the link to the electronic supplementary material.

Supplementary Material 1

## Data Availability

The datasets supporting the present study can be found at https://openneuro.org/datasets/ds000030. The code supporting the analyses is available from the corresponding author upon reasonable request.
