# Supplementary material for: Lag-structure in fMRI across Three Psychiatric Groups: State-dependency and Clinical-behavioral Correlates
Source: Brain Topogr. 2025 Sep 26;38(6):70. doi: 10.1007/s10548-025-01148-5 (PMC12474724; doi:10.1007/s10548-025-01148-5)
Supplement: Supplementary file 1 — Supplementary Material 1 [file 10548_2025_1148_MOESM1_ESM.docx]

# Supplementary Materials

## eMethods 1 - Stopsignal task description

Go stimuli consisted of left- and right-wards pointing arrows to which participants were told to respond by pressing the respective button. For stop trials (25% of total trials), a stop-signal (a 500Hz tone presented through headphones) was presented with a short delay - i.e., stop-signal delay - after the go stimulus appeared and lasted for 250ms. Participants were instructed to respond as quickly and accurately as possible on all trials, but to withhold their response if they heard the stop-signal. They were also instructed that stopping and going were equally important. Performance was then measured through the Stop-Signal Reaction Time (SSRT), based on the horse-race model and considered a critical measure of the cognitive control processes involved in stopping (Logan and Cowan 1984; Verbruggen and Logan 2009). SSRT was computed as the quantile reaction time minus the mean of all stop-signal delay values; longer SSRTs corresponded to worse performances. To yield approximately 50% successful response inhibition for the estimation of stop-signal reaction time (SSRT), the stop-signal delay of each Stop trial was dynamically adjusted (Band et al., 2003).

**Figure S1a** **– Mean framewise displacement (FD) per group – resting state.** **P-values by t-test.**


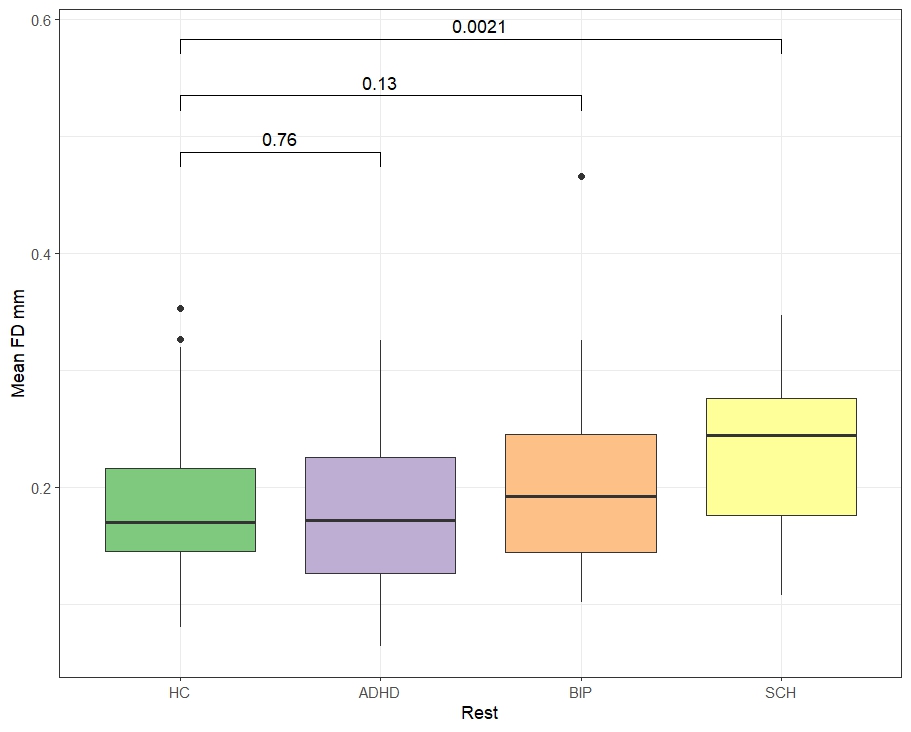


**Figure S1b** **– Mean framewise displacement (FD) per group – task. P-values by t-test.**


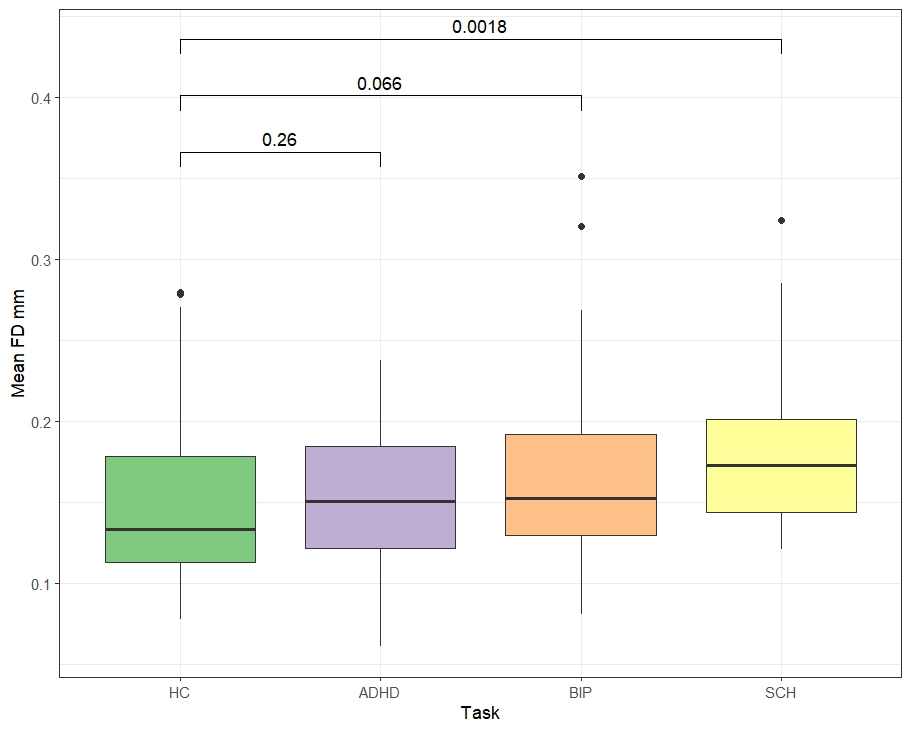


**Figure S2a** **– Mean correlation coefficients for lag-structure – resting state, Pearson rho. P-values by t-test.**


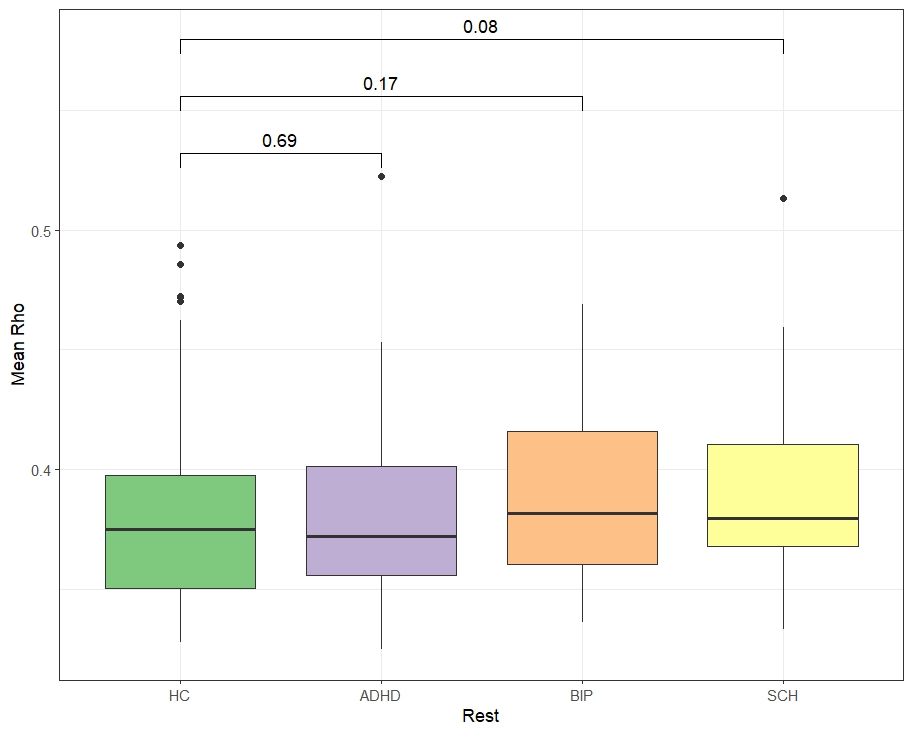


**Figure S2b** **– Mean correlation coefficients for lag-structure – task, Pearson rho. P-values by t-test.**


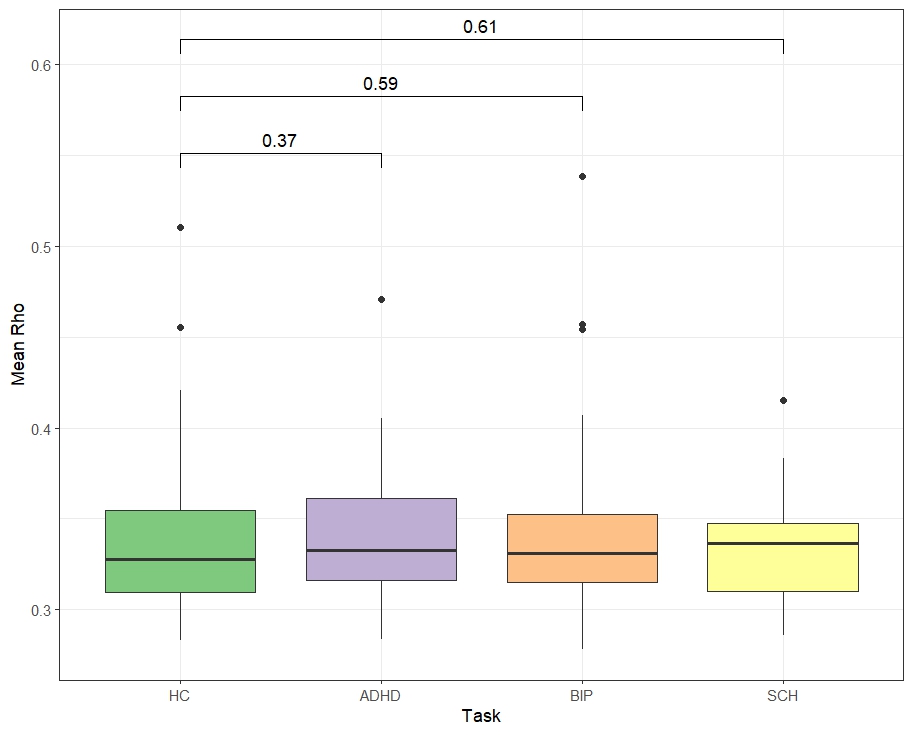


**Figure S3** **– Mean lag-structure per group, resting-state.**

Note: mean lag-structure values per group here shown before masking for gray-matter.


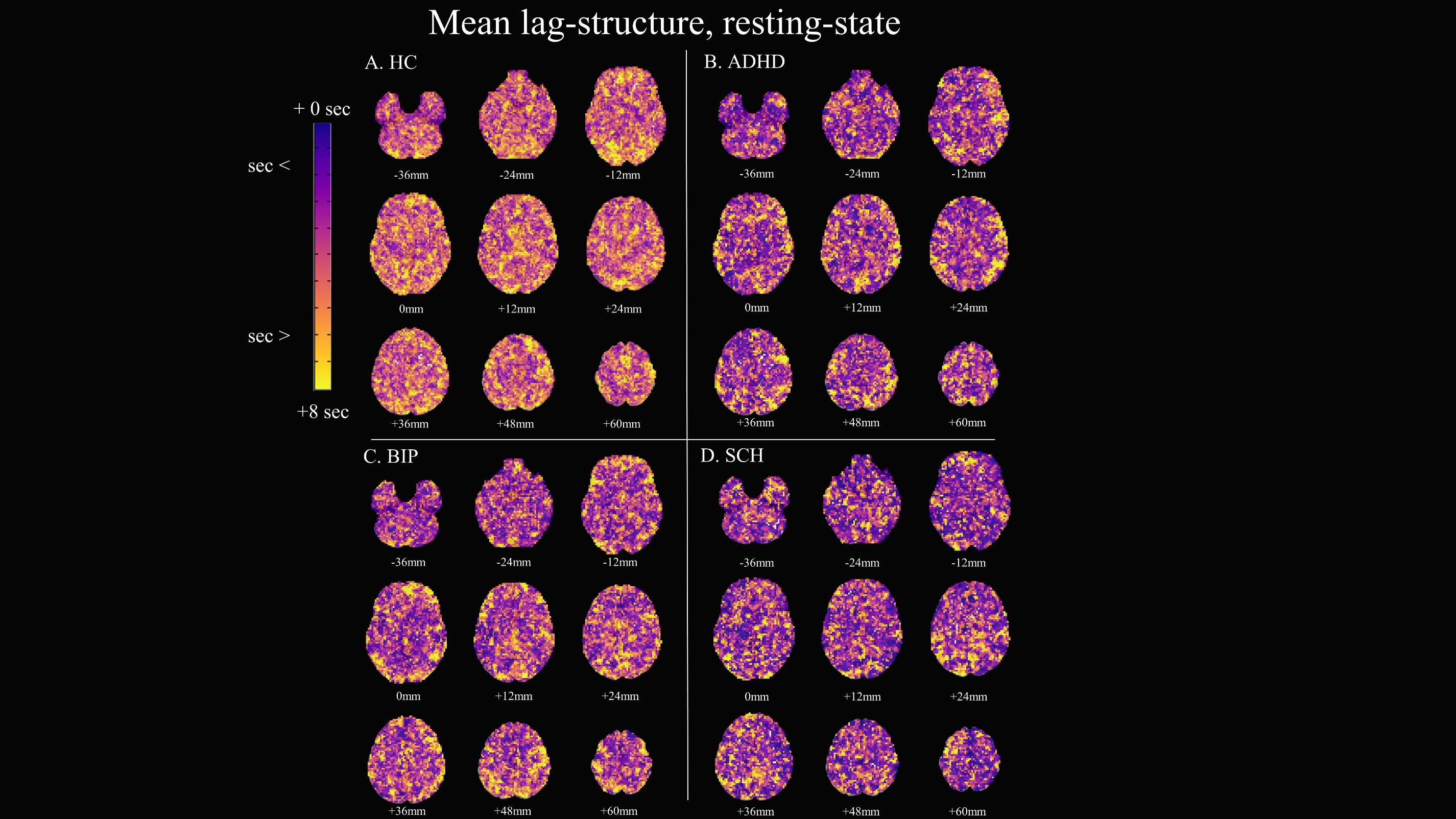


**Figure S4** **– Mean lag-structure per group, resting-state.**

Note: mean lag-structure values per group here shown before masking for gray-matter only.


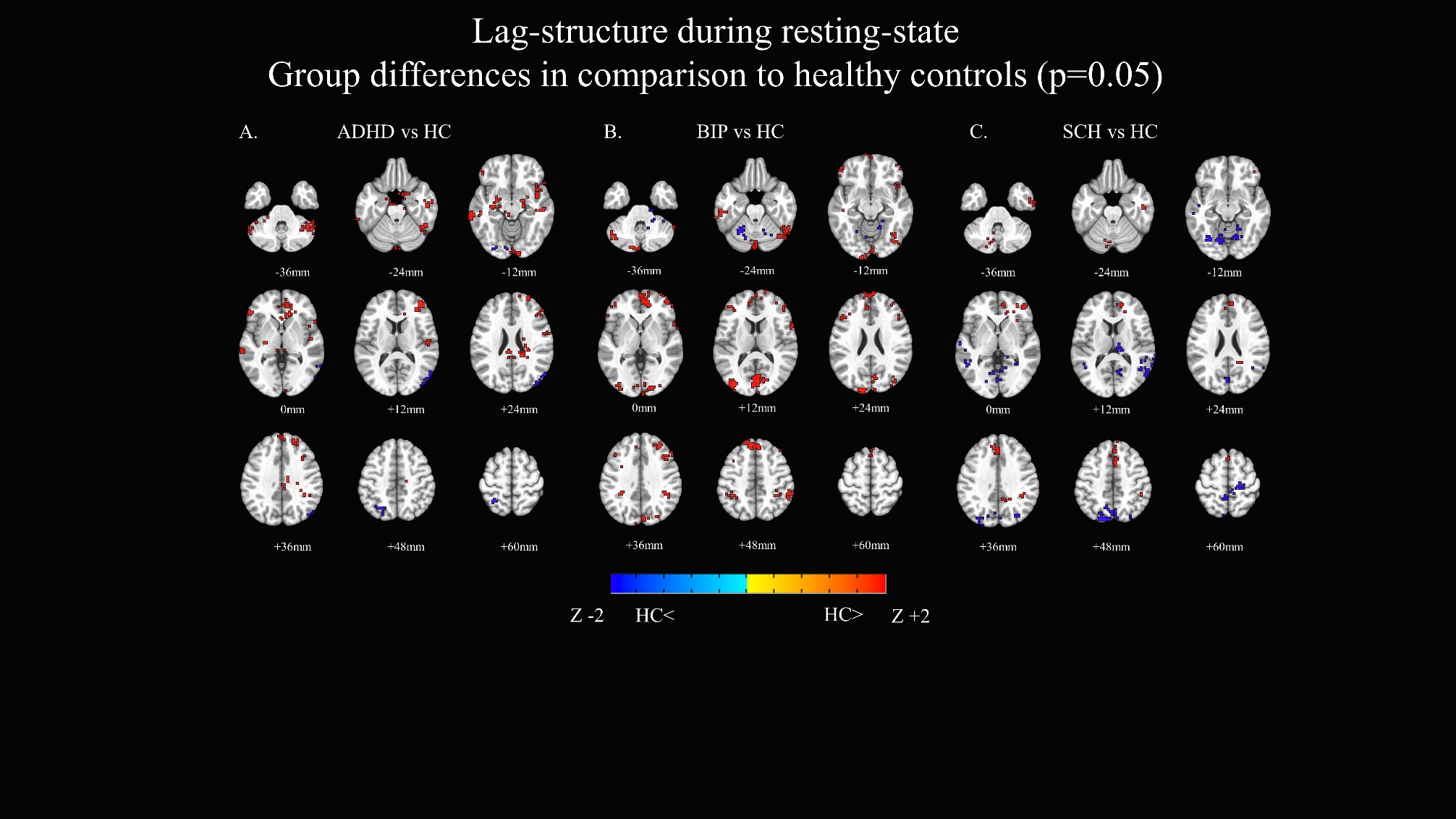


| **Table S1a - Group differences for lag-structure at rest, patients with ADHD vs healthy controls** | | | | |  |
| --- | --- | --- | --- | --- | --- |
| **Cluster number** | **X** | **Y** | **Z** | **Volume (voxels)** | **Z-Score** |
| 1 | -52.5 | +49.5 | -28.5 | 107 | 4.431 |
| **2** | **-52.5** | **+76.5** | **+22.5** | **105** | **-3.983** |
| 3 | -46.5 | +7.5 | -7.5 | 98 | 4.201 |
| 4 | -10.5 | -4.5 | -19.5 | 79 | 3.329 |
| 5 | +28.5 | +16.5 | -10.5 | 78 | 4.088 |
| 6 | -13.5 | -31.5 | -1.5 | 78 | 4.151 |
| 7 | +64.5 | +28.5 | +1.5 | 70 | 4.265 |
| 8 | -10.5 | +19.5 | +34.5 | 66 | 3.647 |
| 9 | +39.5 | +49.5 | -37.5 | 64 | 3.446 |
| 10 | -49.5 | +13.5 | -22.5 | 58 | 3.602 |
| 11 | -7.5 | +97.5 | -7.5 | 54 | 3.407 |
| 12 | -25.5 | -58.5 | +28.5 | 54 | 3.781 |
| 13 | -16.5 | +31.5 | +28.5 | 47 | 3.687 |
| 14 | -4.5 | -49.5 | +43.5 | 43 | 3.910 |
| **15** | **+25.5** | **+64.5** | **+49.5** | **40** | **-3.580** |
| 16 | -37.5 | -22.5 | +40.5 | 39 | 3.842 |
| **17** | **+19.5** | **+91.5** | **-1.5** | **34** | **-3.034** |
| 18 | -40.5 | -49.5 | +13.5 | 32 | 3.253 |
| 19 | -4.5 | +28.5 | +4.5 | 30 | 3.210 |
| 20 | +52.5 | -31.5 | +4.5 | 30 | 3.423 |
| 21 | -58.5 | +1.5 | +19.5 | 30 | 3.255 |
| *Note:* Coordinates represent the peak of activation. Voxels 3 mm x 3 mm x 3 mm. Coordinate Order = RAI. p=0.05 In bold higher values in the group of controls. | | | | | |

| **Table S1b - Group differences for lag-structure at rest, patients with Bipolar Disorder vs healthy controls** | | | | |  |
| --- | --- | --- | --- | --- | --- |
| **Cluster number** | **X** | **Y** | **Z** | **Volume (voxels)** | **Z-Score** |
| 1 | -10.5 | -49.5 | -1.5 | 196 | 3.766 |
| 2 | 1.5 | 82.5 | -16.5 | 144 | 3.802 |
| 3 | -13.5 | 79.5 | 19.5 | 144 | 3.602 |
| 4 | -52.5 | 52.5 | -25.5 | 125 | 4.040 |
| 5 | 10.5 | -40.5 | 49.5 | 105 | 4.059 |
| 6 | -46.5 | -52.5 | 4.5 | 79 | 3.218 |
| 7 | -52.5 | 46.5 | 46.5 | 77 | 3.823 |
| 8 | 34.5 | 91.5 | 1.5 | 75 | 3.788 |
| 9 | 46.5 | 64.5 | -49.5 | 74 | 4.739 |
| 10 | 55.5 | -37.5 | -4.5 | 69 | 3.580 |
| **11** | **-19.5** | **64.5** | **-19.5** | **62** | **-3.010** |
| 12 | -58.5 | -22.5 | -7.5 | 56 | 3.847 |
| **13** | **25.5** | **55.5** | **-22.5** | **52** | **-3.912** |
| 14 | 46.5 | 37.5 | 55.5 | 51 | 3.889 |
| 15 | 52.5 | 28.5 | -25.5 | 48 | 2.868 |
| 16 | 40.5 | -28.5 | 31.5 | 47 | 3.785 |
| 17 | -52.5 | -19.5 | 43.5 | 45 | 3.486 |
| **18** | **-16.5** | **22.5** | **-40.5** | **36** | **-3.183** |
| 19 | 13.5 | 97.5 | 25.5 | 34 | 3.957 |
| 20 | -1.5 | 88.5 | 34.5 | 34 | 3.109 |
| 21 | -31.5 | 88.5 | 28.5 | 33 | 3.187 |
| 22 | -34.5 | -43.5 | 31.5 | 30 | 3.402 |
| *Note:* Coordinates represent the peak of activation. Voxels 3 mm x 3 mm x 3 mm. Coordinate Order = RAI. p=0.05  In bold higher values in the group of controls. | | | | | |

| **Table S1c - Group differences for lag-structure at rest, patients with Schizophrenia vs healthy controls** | | | | |  |
| --- | --- | --- | --- | --- | --- |
| **Cluster number** | **X** | **Y** | **Z** | **Volume (voxels)** | **Z-Score** |
| 1 | 7.5 | -16.5 | 70.5 | 190 | 3.838 |
| **2** | **7.5** | **79.5** | **49.5** | **168** | **-3.368** |
| **3** | **-10.5** | **64.5** | **-7.5** | **140** | **-3.575** |
| **4** | **13.5** | **73.5** | **-7.5** | **112** | **-3.524** |
| **5** | **-55.5** | **64.5** | **13.5** | **102** | **-3.002** |
| **6** | **4.5** | **43.5** | **64.5** | **95** | **-3.716** |
| 7 | 16.5 | 79.5 | -31.5 | 50 | 3.098 |
| **8** | **-22.5** | **46.5** | **73.5** | **48** | **-3.152** |
| 9 | -13.5 | 46.5 | 34.5 | 45 | 3.438 |
| **10** | **-19.5** | **22.5** | **61.5** | **45** | **-3.496** |
| **11** | **52.5** | **19.5** | **-1.5** | **40** | **-2.899** |
| 12 | -40.5 | -43.5 | 7.5 | 39 | 3.324 |
| **13** | **46.5** | **46.5** | **7.5** | **37** | **-3.395** |
| 14 | -31.5 | 37.5 | 34.5 | 36 | 3.411 |
| 15 | -52.5 | 1.5 | -31.5 | 34 | 3.229 |
| 16 | -25.5 | -28.5 | -1.5 | 32 | 3.165 |
| **17** | **-7.5** | **19.5** | **16.5** | **32** | **-3.043** |
| **18** | **-31.5** | **73.5** | **34.5** | **31** | **-3.563** |
| *Note:* Coordinates represent the peak of activation. Voxels 3 mm x 3 mm x 3 mm. Coordinate Order = RAI. p=0.05  In bold higher values in the group of controls. | | | | | |

| **Table S2 - State dependent lag-structure, sample of patients with Schizophrenia** | | | | |  |
| --- | --- | --- | --- | --- | --- |
| **Cluster number** | **X** | **Y** | **Z** | **Volume (voxels)** | **Q-statistics** |
| 1 | +19.5 | +49.5 | -4.5 | 9830 | 17.00 |
| 2 | +46.5 | -28.5 | +34.5 | 147 | 12.25 |
| 3 | -43.5 | +79.5 | -31.5 | 60 | 16.00 |
| 4 | -13.5 | +22.5 | +16.5 | 51 | 13.00 |
| 5 | -40.5 | +49.5 | -49.5 | 47 | 11.00 |
| 6 | -25.5 | +70.5 | -55.5 | 46 | 12.00 |
| 7 | +13.5 | +52.5 | -34.5 | 46 | 9.00 |
| 8 | +25.5 | +37.5 | -34.5 | 41 | 9.00 |
| 9 | +1.5 | -19.5 | +4.5 | 38 | 10.00 |
| 10 | -46.5 | -49.5 | +22.5 | 36 | 11.27 |
| *Note:* Coordinates represent the peak of activation. Voxels 3 mm x 3 mm x 3 mm. Coordinate Order = RAI. p=0.01 | | | | | |

| **Table S3 - State dependent lag-structure and reaction time, stopsignal task** | | | | | |  |
| --- | --- | --- | --- | --- | --- | --- |
| **Sample** | **Cluster number** | **X** | **Y** | **Z** | **Volume (voxels)** | **Z-Score** |
| **ADHD** | 1 | -58.5 | +52.5 | +19.5 | 72 | 2.930 |
|  | 2 | -22.5 | +70.5 | -25.5 | 32 | 3.176 |
|  | 3 | +61.5 | +55.5 | -1.5 | 31 | 3.014 |
| **BIP** | **1** | **+19.5** | **+34.5** | **-25.5** | **75** | **-4.451** |
|  | **2** | **+7.5** | **+100.5** | **-7.5** | **74** | **-4.088** |
|  | **3** | **+61.5** | **+46.5** | **+10.5** | **65** | **-3.864** |
|  | **4** | **+7.5** | **-4.5** | **+46.5** | **62** | **-4.039** |
|  | **5** | **+16.5** | **-37.5** | **-22.5** | **54** | **-3.634** |
|  | **6** | **+43.5** | **+19.5** | **+37.5** | **44** | **-4.206** |
|  | **7** | **+4.5** | **-37.5** | **+43.5** | **44** | **-4.710** |
|  | **8** | **-28.5** | **-10.5** | **+7.5** | **43** | **-4.073** |
|  | **9** | **+37.5** | **+1.5** | **-43.5** | **41** | **-4.137** |
|  | 10 | +10.5 | +94.5 | +4.5 | 35 | 4.510 |
|  | **11** | **+40.5** | **+85.5** | **+13.5** | **35** | **-3.751** |
|  | **12** | **-61.5** | **+16.5** | **+10.5** | **33** | **-3.822** |
|  | 13 | +52.5 | +67.5 | +1.5 | 32 | 4.336 |
|  | **14** | **-43.5** | **+37.5** | **-16.5** | **32** | **-3.674** |
|  | **15** | **+22.5** | **-58.5** | **-13.5** | **31** | **-4.231** |
| **SCH** | **1** | **+40.5** | **+55.5** | **+52.5** | **65** | **-4.086** |
|  | **2** | **+58.5** | **+37.5** | **+52.5** | **61** | **-4.148** |
|  | **3** | **-31.5** | **+61.5** | **+43.5** | **39** | **-3.969** |
|  | **4** | **+19.5** | **+70.5** | **-19.5** | **36** | **-3.788** |
|  | **5** | **+46.5** | **-31.5** | **+37.5** | **33** | **-4.570** |
| *Note:* Coordinates represent the peak of activation. Voxels 3 mm x 3 mm x 3 mm. Coordinate Order = RAI. p=0.05 In bold negative correlations BIP: patients with Bipolar Disorder SCH: patients with Schizophrenia | | | | | |  |
| **Table S4 - State dependent lag-structure and Brief Psychiatric Rating Scale, stopsignal task.** | | | | | |  |
| **Sample** | **Cluster number** | **X** | **Y** | **Z** | **Volume (voxels)** | **Z-Score** |
| **ADHD** | 1 | -52.5 | -37.5 | -13.5 | 309 | 4.030 |
|  | 2 | +52.5 | +58.5 | +4.5 | 160 | 3.808 |
|  | 3 | -55.5 | +19.5 | -4.5 | 138 | 3.684 |
|  | 4 | -58.5 | +37.5 | +16.5 | 133 | 4.378 |
|  | 5 | -58.5 | +67.5 | +1.5 | 99 | 3.947 |
|  | 6 | +22.5 | -4.5 | -16.5 | 60 | 3.546 |
|  | 7 | -25.5 | +85.5 | +28.5 | 60 | 3.359 |
|  | 8 | -16.5 | -19.5 | +64.5 | 55 | 3.775 |
|  | 9 | +43.5 | -16.5 | +4.5 | 52 | 3.900 |
|  | **10** | **-58.5** | **+1.5** | **+4.5** | **47** | **-3.401** |
|  | **11** | **+36.5** | **-10.5** | **+13.5** | **46** | **-3.873** |
|  | 12 | -67.5 | +10.5 | +28.5 | 43 | 3.550 |
|  | 13 | -37.5 | +76.5 | +4.5 | 42 | 3.337 |
|  | 14 | +28.5 | +34.5 | -4.5 | 36 | 3.487 |
|  | 15 | -40.5 | -10.5 | +25.5 | 31 | 3.269 |
|  | **16** | **+31.5** | **+43.5** | **+70.5** | **30** | **-3.421** |
| **BIP** | 1 | -58.5 | -4.5 | -10.5 | 48 | 4.500 |
|  | 2 | -46.5 | +58.5 | -46.5 | 44 | 3.307 |
|  | 3 | -28.5 | -4.5 | -19.5 | 43 | 3.608 |
|  | 4 | +7.5 | +52.5 | -13.5 | 41 | 3.453 |
|  | 5 | -52.5 | +7.5 | +49.5 | 31 | 3.833 |
| **SCH** | **1** | **+34.5** | **+79.5** | **-22.5** | **68** | **-3.142** |
|  | 2 | +19.5 | -31.5 | +37.5 | 39 | 3.311 |
| *Note:* Coordinates represent the peak of activation. Voxels 3 mm x 3 mm x 3 mm. Coordinate Order = RAI. p=0.05. In bold negative correlations BIP: patients with Bipolar Disorder SCH: patients with Schizophrenia | | | | | |  |

**Figure S4** **– Global Signal Regression (GSR), group differences, resting-state**


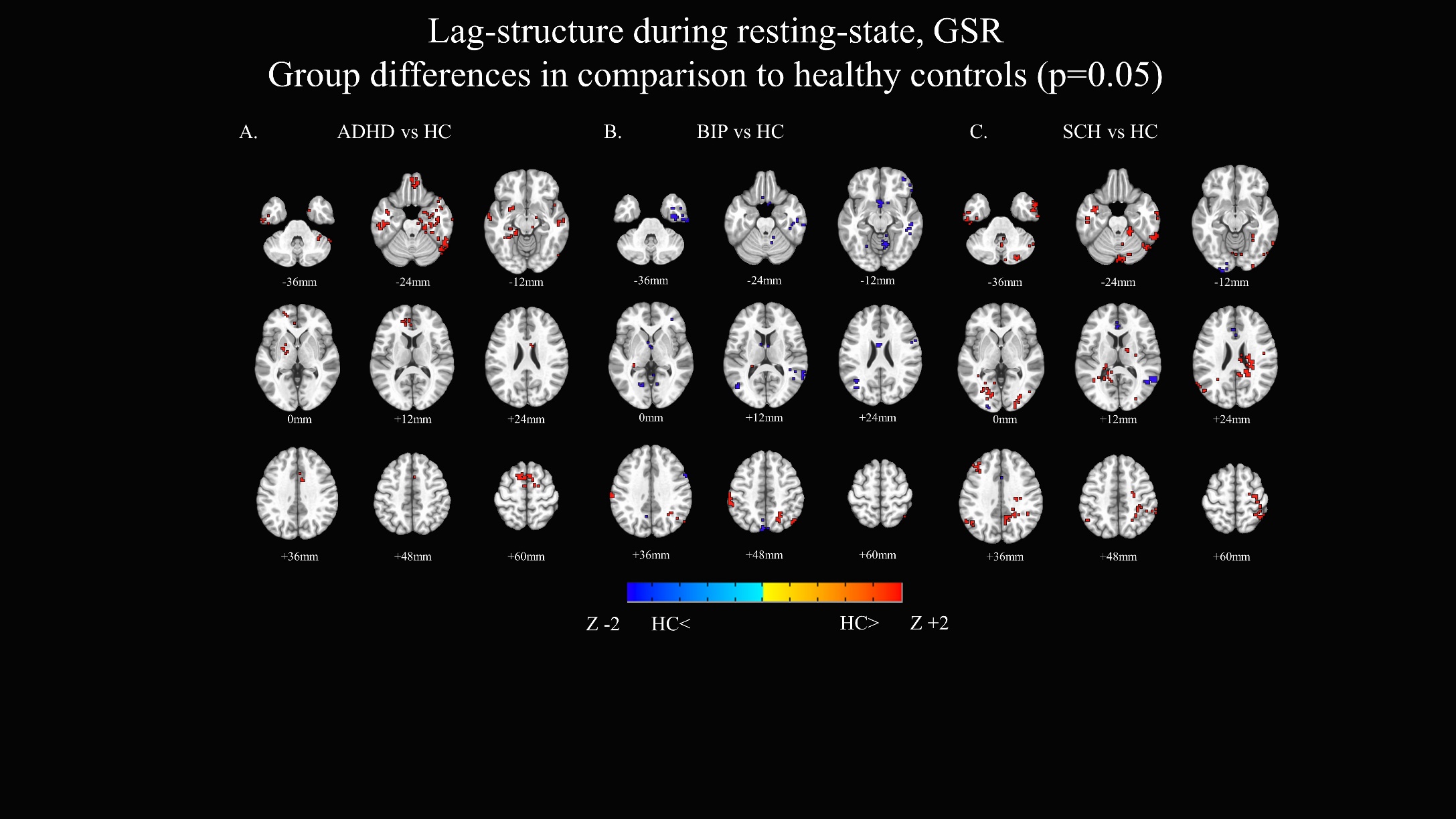


| **Table S5a - Global Signal Regression (GSR), group differences for patients with ADHD vs healthy controls** | | | | |  |
| --- | --- | --- | --- | --- | --- |
| **Cluster number** | **X** | **Y** | **Z** | **Volume (voxels)** | **Z-Score** |
| **1** | -1.5 | -13.5 | 67.5 | 141 | 3.851 |
| **2** | -67.5 | 16.5 | -16.5 | 139 | 3.719 |
| **3** | -55.5 | 52.5 | -25.5 | 99 | 3.999 |
| **4** | 64.5 | 7.5 | -7.5 | 92 | 3.758 |
| **5** | -16.5 | 7.5 | -13.5 | 65 | 3.901 |
| **6** | 22.5 | 1.5 | 1.5 | 52 | 3.928 |
| **7** | 10.5 | -37.5 | 10.5 | 50 | 3.902 |
| **8** | 28.5 | 43.5 | -7.5 | 42 | 4.111 |
| **9** | -19.5 | 7.5 | 67.5 | 40 | 3.969 |
| **10** | -7.5 | -46.5 | -22.5 | 30 | 3.594 |
| *Note:* Coordinates represent the peak of activation. Voxels 3 mm x 3 mm x 3 mm. Coordinate Order = RAI. p=0.05  In bold higher values in the group of controls. | | | | | |

| **Table S5b - Global Signal Regression (GSR), group differences for patients with Bipolar Disorder vs healthy controls** | | | | |  |
| --- | --- | --- | --- | --- | --- |
| **Cluster number** | **X** | **Y** | **Z** | **Volume (voxels)** | **Z-Score** |
| **1** | **-46.5** | **31.5** | **-16.5** | **93** | **-3.207** |
| **2** | **-10.5** | **55.5** | **-10.5** | **76** | **-3.095** |
| **3** | 61.5 | 28.5 | 49.5 | 70 | 3.912 |
| **4** | **-1.5** | **-13.5** | **-7.5** | **67** | **-3.529** |
| **5** | -31.5 | 58.5 | 55.5 | 59 | 4.020 |
| **6** | **46.5** | **70.5** | **16.5** | **54** | **-3.433** |
| **7** | **-40.5** | **-46.5** | **-7.5** | **50** | **-3.098** |
| **8** | **1.5** | **79.5** | **49.5** | **45** | **-3.012** |
| **9** | **-49.5** | **49.5** | **10.5** | **42** | **-2.862** |
| **10** | -46.5 | 58.5 | 55.5 | 38 | 3.260 |
| **11** | **13.5** | **64.5** | **1.5** | **38** | **-2.652** |
| **12** | **-52.5** | **10.5** | **-34.5** | **36** | **-3.057** |
| **13** | 31.5 | 31.5 | 7.5 | 33 | 3.849 |
| **14** | **-58.5** | **-7.5** | **+34.5** | **33** | **-2.746** |
| *Note:* Coordinates represent the peak of activation. Voxels 3 mm x 3 mm x 3 mm. Coordinate Order = RAI. p=0.05.  In bold higher values in the group of controls. | | | | | |

| **Table S5c - Global Signal Regression (GSR), group differences for patients with Schizophrenia vs healthy controls** | | | | |  |
| --- | --- | --- | --- | --- | --- |
| **Cluster number** | **X** | **Y** | **Z** | **Volume (voxels)** | **Z-Score** |
| **1** | -22.5 | 52.5 | 31.5 | 221 | 4.681 |
| **2** | -19.5 | 7.5 | 67.5 | 114 | 4.893 |
| **3** | -25.5 | 25.5 | 25.5 | 103 | 5.406 |
| **4** | 52.5 | 58.5 | 46.5 | 77 | 4.107 |
| **5** | 40.5 | 52.5 | 13.5 | 69 | 4.493 |
| **6** | -22.5 | 82.5 | -37.5 | 61 | 4.179 |
| **7** | -58.5 | -4.5 | -34.5 | 57 | 3.348 |
| **8** | -52.5 | 64.5 | -25.5 | 57 | 3.950 |
| **9** | **-1.5** | **-22.5** | **22.5** | **56** | **-3.043** |
| **10** | 37.5 | 13.5 | -31.5 | 52 | 3.110 |
| **11** | -1.5 | 85.5 | -22.5 | 50 | 3.539 |
| **12** | 46.5 | -34.5 | 37.5 | 50 | 3.393 |
| **13** | -58.5 | 49.5 | -25.5 | 45 | 3.861 |
| **14** | **-49.5** | **49.5** | **10.5** | **45** | **-2.709** |
| **15** | -28.5 | 94.5 | -10.5 | 44 | 4.536 |
| **16** | 16.5 | 82.5 | 1.5 | 44 | 3.840 |
| **17** | 52.5 | 16.5 | -28.5 | 41 | 3.939 |
| **18** | 13.5 | 58.5 | -46.5 | 35 | 3.240 |
| **19** | -19.5 | 37.5 | -22.5 | 32 | 3.618 |
| **20** | **19.5** | **88.5** | **-19.5** | **31** | **-2.709** |
| *Note:* Coordinates represent the peak of activation. Voxels 3 mm x 3 mm x 3 mm. Coordinate Order = RAI. p=0.05 | | | | | |

In bold higher values in the group of controls.

**Figure S5** **– Global Signal Regression (GSR), the gradient of severity was noticeable irrespective of GSR or noGSR (see Figure 1).**


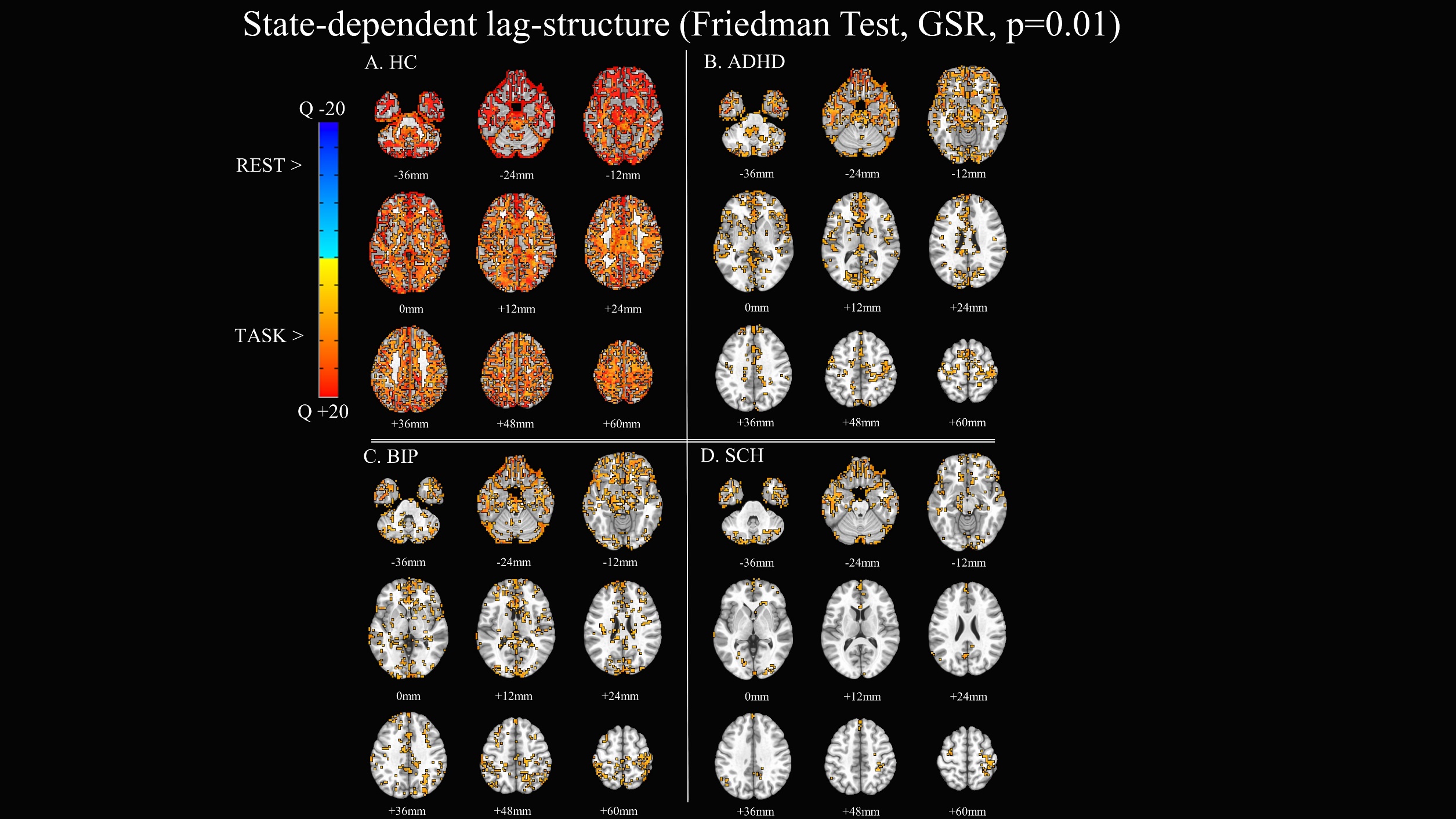


**Figure S6** **- Motion Effect, correlation between state dependent lag-structure and motion at rest (as measured by mean FD). Only Schizophrenia showed significant results. No other group showed significant correlations with motion and state dependent lag-structure.**

1. *Schizophrenia*


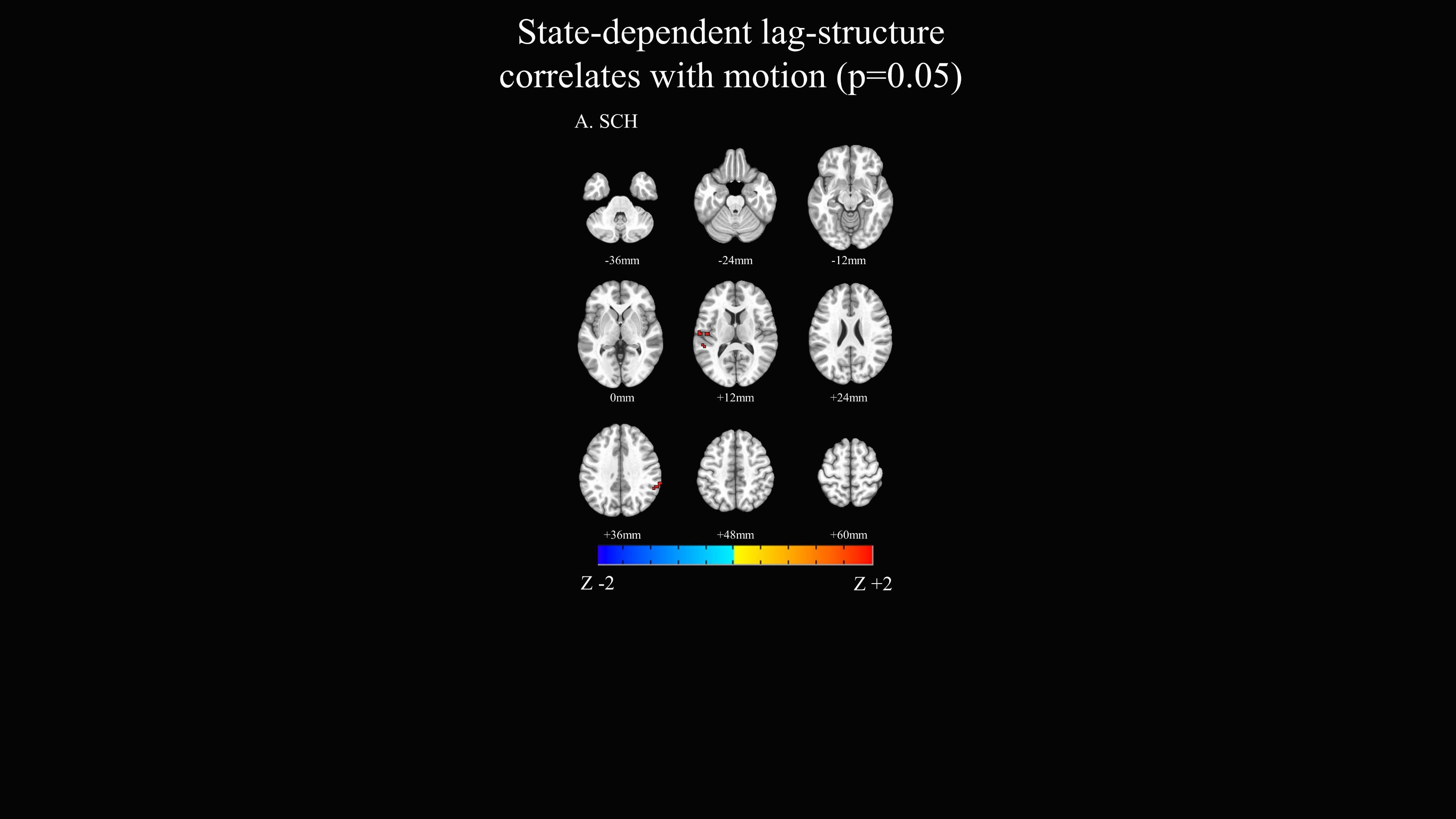


| **Table S6 - Motion effect on lag-structure, results for sample of patients with Schizophrenia** | | | | |  |
| --- | --- | --- | --- | --- | --- |
| **Cluster number** | **X** | **Y** | **Z** | **Volume (voxels)** | **Z-Score** |
| **1** | +46.5 | +16.5 | +10.5 | 49 | 3.281 |
| **2** | -61.5 | +40.5 | +43.5 | 31 | 3.117 |
| *Note:* Coordinates represent the peak of activation. Voxels 3 mm x 3 mm x 3 mm. Coordinate Order = RAI. p=0.05 | | | | | |

# 
